# Potential Fossilized Sulfide-Oxidizing Bacteria in the Upper Miocene Sulfur-Bearing Limestones From the Lorca Basin (SE Spain): Paleoenvironmental Implications

**DOI:** 10.3389/fmicb.2019.01031

**Published:** 2019-05-21

**Authors:** Federico Andreetto, Francesco Dela Pierre, Luis Gibert, Marcello Natalicchio, Simona Ferrando

**Affiliations:** ^1^Dipartimento di Scienze della Terra, Università degli Studi di Torino, Turin, Italy; ^2^Departament de Mineralogia, Petrologia i Geologia Aplicada, Universitat de Barcelona, Barcelona, Spain

**Keywords:** microbial filaments, fecal pellets, sulfide-oxidizing bacteria, Lorca Basin, evaporitic limestones

## Abstract

The sulfur-bearing limestones interbedded in the upper Miocene diatomaceous sediments (Tripoli Formation) of the Lorca Basin (SE Spain) are typified, as other Mediterranean coeval carbonate and gypsum deposits, by filamentous, circular and rod-shaped microstructures of controversial origin. These features have been interpreted both as fecal pellets of brine shrimps and/or of copepods, remains of algae or cyanobacteria and fossilized sulfide-oxidizing bacteria. To shed light on their origin, a multidisciplinary study including optical, UV and scanning electron microscopy, Raman microspectroscopy, and geochemical (carbon and oxygen stable isotopes) analyses has been carried out on three carbonate beds exposed along the La Serrata ridge. The different composition of the filamentous and circular objects with respect to the rod-shaped microstructures suggest that the former represent remains of bacteria, while the latter fecal pellets of deposit- or suspension-feeder organisms. Size and shape of the filamentous and circular microfossils are consistent with their assignment to colorless sulfide-oxidizing bacteria like *Beggiatoa* (or *Thioploca)* and *Thiomargarita*, which is further supported by the presence, only within the microfossil body, of tiny pyrite grains. These grains possibly result from early diagenetic transformation of original sulfur globules stored by the bacteria, which are a diagnostic feature of this group of prokaryotes. The development of microbial communities dominated by putative sulfide-oxidizing bacteria at Lorca was favored by hydrogen sulfide flows generated through degradation of organic matter by sulfate-reducing bacteria thriving in underlying organic-rich sediments.

## Introduction

The upper Miocene sedimentary record of the Mediterranean region comprises peculiar carbonate rocks typified by molds of evaporitic minerals (mainly gypsum) and by elemental sulfur nodules. These rocks have been described in the Messinian succession of Sicily (the so-called Calcare di Base, e.g., Decima et al., 1988; Rouchy and Caruso, 2006; Guido et al., 2007; Oliveri et al., 2010; Ziegenbalg et al., 2010; Birgel et al., 2014; Caruso et al., 2015; Perri et al., 2017) and in the upper Miocene successions of Spanish intramontane basins (e.g., Granada Basin: García-Veigas et al., 2015; Lorca Basin: Rouchy et al., 1998; Hellín Basin: Lindtke et al., 2011; Las Minas-Camarillas Basin: Ortí et al., 2014) of the Betic Cordillera. A common feature of these rocks is the peloidal and microsparitic texture, believed to result from the metabolic activity of sulfate-reducing bacteria which, by degrading different organic substrates (e.g., particulate organic matter, methane, and crude oil), promote an increase of alkalinity and the precipitation of a wide array of carbonate minerals (aragonite, calcite, and dolomite) (Feely and Kulp, 1957; Anadón et al., 1992; Machel, 2001; Baumgartner et al., 2006; Jørgensen and Kasten, 2006; Ziegenbalg et al., 2010). The ^13^C-depleted signature of these minerals is a proof of the bacterial involvement in carbonate precipitation (e.g., Ziegenbalg et al., 2010; Natalicchio et al., 2012). The oxidation of hydrogen sulfide produced by microbial sulfate reduction is considered as responsible for the formation of native sulfur during early (syngenetic) or late (epigenetic) diagenetic processes (Ruckmick et al., 1979; Wessel, 1994; Ziegenbalg et al., 2010). Another common feature of the upper Miocene limestones is the almost completely absence of macrofossils, which is believed to result from harsh environmental conditions (e.g., hypersalinity, anoxia) lethal for most eukaryotes (e.g., Bellanca et al., 2001; Blanc-Valleron et al., 2002). In contrast, these rocks are typified by peculiar assemblages of filamentous, circular and rod-shaped microstructures of controversial origin: remains of algae (Vai and Ricci Lucchi, 1977) or bacteria (Oliveri et al., 2010; Dela Pierre et al., 2012, 2014; Caruso et al., 2015; Perri et al., 2017), fecal pellets of brine shrimps (*Artemia salina*) adapted to hypersaline conditions (e.g., Schreiber, 1978; Natalicchio et al., 2013; García-Veigas et al., 2015) or of copepods (Guido et al., 2007) thriving in a normal marine water column, thus excluding the presence of hypersaline conditions. The distinction of the predominating group of precursor microorganisms beyond these enigmatic structures is fundamental to assess the environmental conditions in the water column and on the bottom of the basin during deposition and early diagenetic phase. This paper addresses the study of the morphologically distinct microstructures preserved in sulfur-bearing limestones from the upper Miocene succession of the Lorca Basin (SE Spain). Their investigation with a multidisciplinary approach, which combines petrographic (optical, electronic, and fluorescence microscopy), spectroscopic (micro-Raman) and geochemical (C and O stable isotopes) techniques, allowed to shed light on the precursor organisms, and consequently, on the environmental conditions during deposition and early diagenesis.

## Geological Setting

**FIGURE 1:**
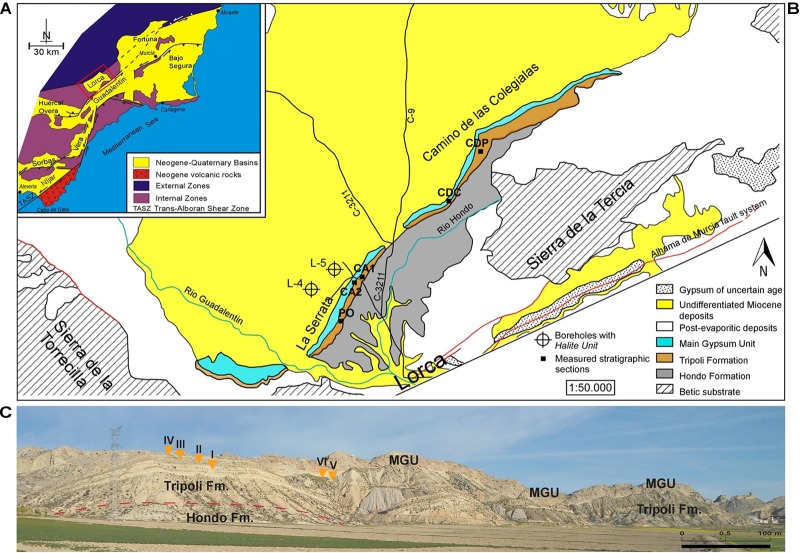
**(A)** Schematic geological map of the eastern Betic Cordillera (modified after Soria et al., 2014). The red rectangle indicates the location of the Lorca Basin. **(B)** Geological map of the south-western sector of the Lorca Basin (modified from Kampschuur et al., 1972), with location of the studied stratigraphic sections. PO, Pig section; CA1, Eastern Las Minas Volcas section; CA2, Western Las Minas Volcas section; CDC, Cortijo de las Colegialas section; CDP, Cortijo del Pozuelo section. **(C)** Panoramic view of the La Serrata ridge. Note the sharp contact between the Tripoli Fm. and the Main Gypsum Unit (MGU). Orange triangles point the position of the sulfur-bearing limestones (I–VI).

The Lorca Basin is a post-orogenic pull-apart basin located in the eastern Betic Cordillera (Montenat et al., 1990; Guillén-Mondéjar et al., 1995; Turell et al., 1997; Rouchy et al., 1998; Figure 1A). This basin is filled with up to 1500 m of Tortonian/Pliocene sediments unconformably overlying the metamorphic basement of the Betic Cordillera (Geel, 1976; Montenat et al., 1990; Benali et al., 1995; Guillén-Mondéjar et al., 1995; Turell et al., 1997; Rouchy et al., 1998; Vennin et al., 2004). The upper Miocene succession (Figure 1B), entirely exposed along the NE-SW trending La Serrata ridge (Figure 1C), begins with open marine fossiliferous marls (Hondo Formation), followed by an alternation of marls and finely laminated diatomites. This unit, referred to as Varied Member (Geel, 1976) or Tripoli Formation (Fm.) (Rouchy et al., 1998, term that is used in this paper), records the progressive isolation of the Lorca Basin from the Mediterranean Sea in response to the tectonic uplift of the Betic units (Krijgsman et al., 2000, 2006; Garcés et al., 2001). This trend toward more restricted conditions culminated with deposition of two evaporitic units consisting of gypsum (Main Gypsum Unit; Rouchy et al., 1998) and halite (García-Veigas, 1993; Ayora et al., 1994; García-Veigas et al., 1994). These evaporitic units document the total isolation of the basin and the subsequent establishment of continental conditions (Montenat et al., 1990; Ortí et al., 1993; Rouchy et al., 1998; Krijgsman et al., 2000; Playà et al., 2000). The age of the Tripoli Fm. and overlying evaporites is still discussed. According to Rouchy et al. (1998), both these units are of Messinian age and are coeval with analogous Mediterranean successions deposited immediately prior and during the Messinian salinity crisis (5.97–5.33 Ma; Manzi et al., 2013), when the Mediterranean was turned into a giant salina (e.g., Rouchy and Caruso, 2006; CIESM, 2008; Roveri et al., 2014). Krijgsman et al. (2000) have instead suggested a late Tortonian age for the Tripoli Fm. and the evaporites, deposition of which was triggered by the early tectonic isolation of the internal Betic basins (including Lorca) during the so-called Tortonian salinity crisis of the eastern Betics.

### The Tripoli Formation

**FIGURE 2:**
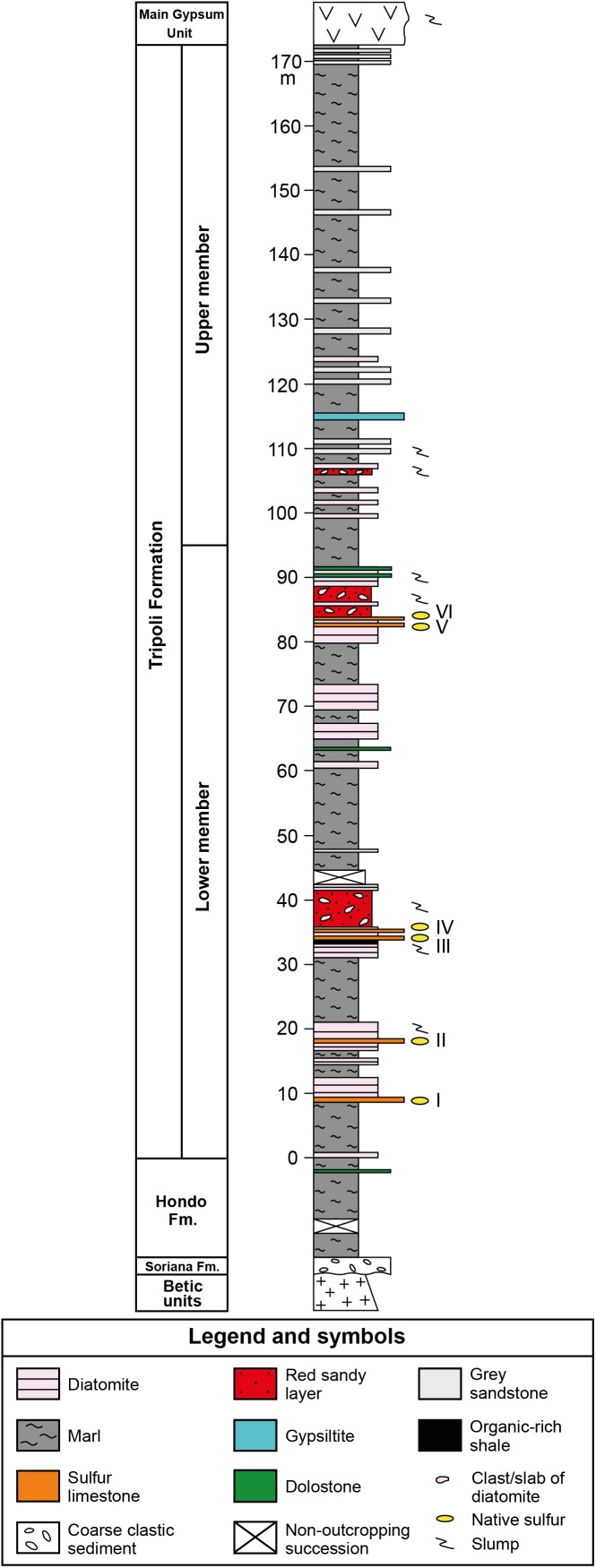
Composite stratigraphic column of the Tripoli Fm., resulting from the physical correlation of the Pig (PO) and Las Minas Volcas (CA1 and CA2) sections (location of the sections in Figure 1B). Roman numbers indicate the sulfur-bearing limestone layers.

The Tripoli Fm. has been further subdivided into two members on the basis of the presence/absence of biosiliceous deposits: the lower member, about 120 m thick in the basin depocenter, shows a distinct lithological cyclicity expressed by the alternation of marls and diatomaceous layers (Figure 2). According to Krijgsman et al. (2000), the lithological cyclicity is controlled by precession-driven climate changes. In the Lorca Basin, six layers of sulfur-bearing limestones are interbedded in the lower member of the Tripoli Fm. (Rouchy et al., 1998). These layers make a lateral transition, toward the NE margin of the basin, to an equal number of alabastrine gypsum beds (Rouchy et al., 1998). The upper member is instead thinner and mostly composed of marls and sandstones (Figure 2).

In previous studies (Benali et al., 1995; Russell et al., 1997; Rouchy et al., 1998) the sulfur-bearing limestones were believed to result from microbially-driven diagenetic processes in an evaporitic and anoxic environment recording sea-level lowstand phases. The supposed evaporitic conditions are based on the presence of abundant calcite pseudomorphs after gypsum and on the chicken-wire structure (Rouchy et al., 1998) of the correlative secondary gypsum levels. These features are thought to indicate deposition in very shallow-water and sabkha environment with episodes of subaerial exposure (Benali et al., 1995; Russell et al., 1997; Rouchy et al., 1998). The influence of sulfate-reducing bacteria was instead suggested on the basis of the replacement of gypsum by carbonates with negative δ^13^C values and the abundant elemental sulfur nodules included in the carbonates (Rouchy et al., 1998).

## Materials and Methods

Field studies of the lithological and sedimentological features of the sulfur-bearing limestones and hosting sediments were performed on four stratigraphic sections located along the La Serrata and the Camino de las Colegialas ridges (Figure 1B). Three samples were taken from each layer of sulfur-bearing limestone and two samples from the under- and overlying sediments. Each sample was split into two parts, one used for petrographical and mineralogical analyses, the other for stable isotope analyses.

Twenty standard thin sections from the most representative samples were studied under an optical microscope in both transmitted and reflected light. Fluorescence intensity was evaluated using a Nikon microscope equipped with a B2-filter block (illumination source with an excitation wavelength of 395–440 nm). Scanning electron microscopy (SEM) analyses were performed on six stubs for morphological analyses and on twelve polished carbon-coated thin sections for semiquantitative elemental analyses and backscattered electron imagery using an environmental scanning electron microscope (ESEM FEI-Quanta 2000) coupled with a backscattered electron detector (BSED) (Centres Científics i Tecnològics, Universitat de Barcelona) and a JSM-IT300LV SEM equipped with an energy-dispersive EDS Oxford Instrument Link System microprobe (Department of Earth Sciences, University of Torino).

*In situ* micro-Raman spectra from the same thin sections used for petrographic observations were acquired using the integrated micro/macro-Raman LABRAM HRVIS (Horiba Jobin Yvon Instruments) of the Interdepartmental Center “G. Scansetti” (Department of Earth Sciences, University of Torino, Italy). Excitation line at 532 nm (solid-state Nd laser and 80 mW of emission power) was used, with slit at 300 μm and a grating of 600 grooves/mm; the corresponding spectral resolution was 4 cm^-1^. Each spectrum was collected in confocal setting with a hole of 200 μm and the laser was focused on the sample using an Olympus BX41 microscope with an objective 100 × (spot size resolution of ca. 1 × 1 × 3 μm). One to five accumulations in the time span of 2–20 s were collected for each spectrum and D1, D2, or D3 filters have been alternatively inserted. Calibration was performed using the 520.6 cm^-1^ Si band.

Carbon (δ^13^C) and oxygen (δ^18^O) stable isotope analyses of the main carbonate phases have been performed on each bed of sulfur-bearing limestone. The carbonate samples were dissolved with phosphoric acid and the resulting CO_2_ was measured eight times to calculate average outcomes and standard deviations. CO_2_ extraction was done in a Thermo Finnigan Carbonate Kiel Device III, which reproduces in an automated way a modified version of the McCrea method (McCrea, 1950). Carbonate is attacked with 100% phosphoric acid at 70°C, with a 4 min reaction time. The Carbonate Device is coupled to an isotope ratio mass spectrometer Thermo Finnigan MAT-252, where the produced CO_2_ is analyzed on-line. Results were calibrated with secondary standards, traceable to NBS-18 and NBS-19 international standards. The analytical error was less than ±0.04‰ for δ^13^C and ±0.06‰ for δ^18^O.

## Results

### Field Observations

**FIGURE 3:**
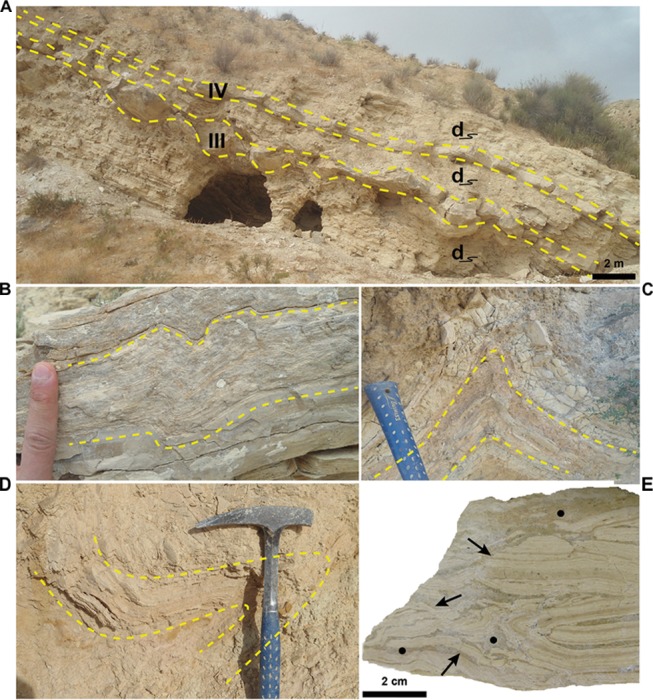
Field and hand-sample features. **(A)** Outcrop view of layers III and IV (yellow dashed lines) and interbedded slumped diatomaceous sediments (d). Note the undulated aspect of the lower and upper contacts, especially of layer III. **(B,C)** Close up of slumped, diatomaceous layers. **(D)** A slab of a deformed diatomaceous layers (yellow dashed lines) incorporated in a reddish sandy bed emplaced by gravity flows. Hammer for scale. **(E)** Polished slab of the of the upper part of layer I. Black arrows indicate deformed diatomaceous clasts floating in a micrite matrix (black circle).

The six layers of sulfur-bearing limestones, ranging in thickness from 10 to 60 cm, are interbedded in the lower member of the Tripoli Fm. (Figure 2). The lower and upper contacts with hosting sediments are sharp and undulated (Figure 3A). The studied layers are interbedded to slumped diatomaceous and marly sediments (Figure 3B,C), frequently (layers III to VI; see Figure 2) followed by reddish sandy layers rich in plant remains and clasts or deformed slabs of diatomaceous strata (Figure 3D), emplaced by gravity flows. Bed I contains in the upper part cm- to dm-sized clasts of diatomites (Figure 3E) and fragments of coralline algae, suggesting that it is the product of gravity flows sourced by erosion of marginal shelf deposits.

### Petrographic Observations

**FIGURE 4:**
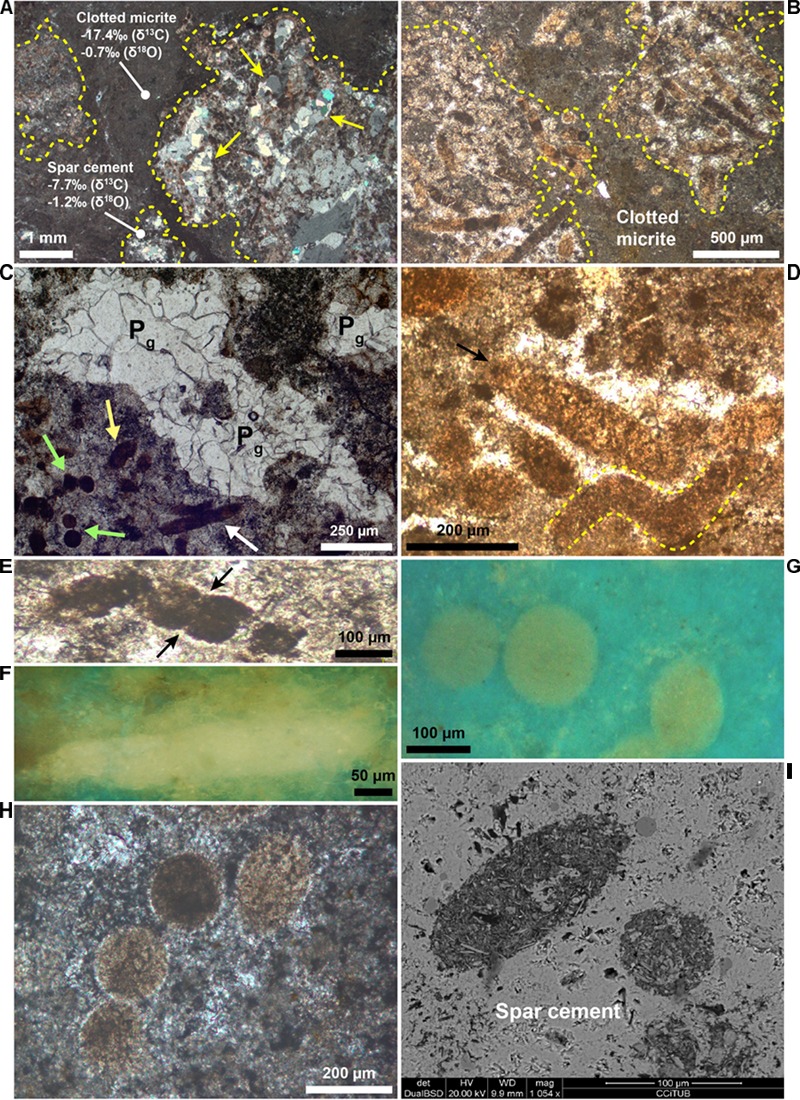
Petrography of the filamentous, circular, and rod-shaped microstructures. **(A**,**B)** Photomicrographs of layer I cut perpendicular (**A**, crossed polars) and parallel (**B**, transmitted light) to the bedding, showing some filaments-bearing patches (dashed yellow lines) surrounded by clotted micrite. Note in **(A)** the calcite pseudomorphs after gypsum (yellow arrows) within a filament-bearing patch. Carbon and oxygen isotope values of clotted micrite and spar calcite around the filaments are shown. **(C)** Photomicrograph (transmitted light) showing a cluster of filamentous (white arrow), circular (green arrows), and rod-shaped (yellow arrow) microstructures. Calcite pseudomorphs after gypsum (P_g_) are visible in the center. **(D)** Photomicrograph (transmitted light) of filamentous microstructures (arrow and yellow dashed lines), locally with a curved morphology (yellow dashed lines). **(E)** Photomicrograph (transmitted light) of a segmented and curved filament. The arrows indicate the internal segmentation. **(F)** UV light photomicrograph of a filament. Note its intense yellow fluorescence. **(G)** Photomicrograph in UV light of a cluster of circular microfossils. Note their yellowish epifluorescence, very similar to the filaments in panel **(F)**. **(H)** Alignment of four circular microstructures with a similar diameter (crossed polars). **(I)** Backscatter SEM image of two rod-shaped features. Note the sharp compositional contrast with the surrounding low-Mg calcite cement.

Layers I, II, and V (Figure 2) are typified by dense aggregates of filamentous, circular and rod-shaped microstructures (Figure 4A–C). In the remaining layers (III, IV, and VI; Figure 2) these features are badly preserved and less clear.

The filaments are more than 500–600 μm long, with a cross-sectional diameter up to 80 μm (Figure 4D) and rather uniform throughout their length. They are frequently curved (Figure 4D) and positioned with their long axis both perpendicular and parallel to the bedding. Some filaments are typified by an internal transversal segmentation (Figure 4E). In transmitted light filaments appear dark black (Figure 4E) to light brown (Figure 4D). The color depends on the relative content of their two main components, i.e., dark micrite and light brown microsparitic calcite. The former consists of an aggregate of micrometer to nanometer-sized calcite crystals typified by a strong yellow fluorescence (Figure 4F) reflecting the incorporation of organic matter, while the latter owns a blue-green fluorescence. The circular features, 70–120 μm across, show a circular to slightly elliptical shape (Figure 4G,H) and occur either isolated (Figure 4G) or grouped to form “chains” up to 800 μm long (Figure 4H). The circular objects are characterized by the same composition and yellow fluorescence (Figure 4G, 5D) as the filaments.

**FIGURE 5:**
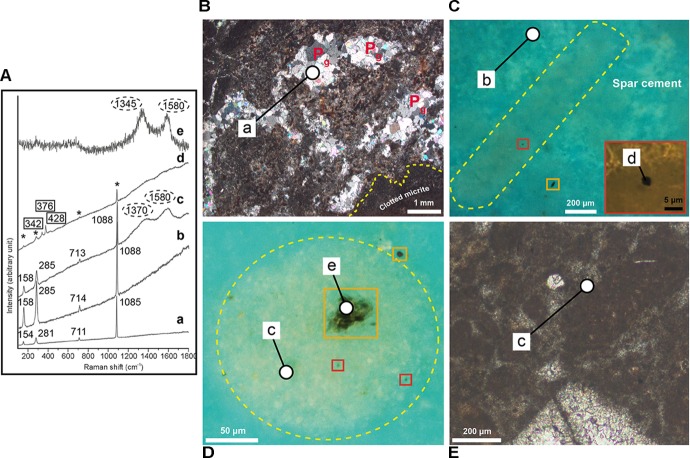
**(A)** From bottom to top, representative micro-Raman spectra. (a) Spectrum of sparry calcite (unconfined numbers) replacing primary gypsum crystals. (b) Spectrum of low-Mg spar-calcite (unconfined numbers) that represents the cement embedding the filamentous and circular microstructures. (c) Mixed spectrum of low-Mg calcite (unconfined numbers) with poorly organized carbonaceous material (circles) collected from calcite inside a circular microstructure and in the clotted micrite. (d) Mixed spectrum of pyrite (rectangles), low Mg-calcite (asterisks) and poorly organized carbonaceous material (unlabelled) collected from a small pyrite grain inside a filament. (e) Spectrum of poorly organized carbonaceous material (circles) collected from an opaque grain inside a circular object. **(B)** Photomicrographs in transmitted light (crossed polars) of carbonate layer V cut perpendicular to the bedding, showing some calcite pseudomorphs after gypsum (P_g_) within filament-bearing patches (dashed yellow lines) surrounded by clotted micrite. **(C)** Photomicrograph in UV light of a filaments (dashed yellow lines). Orange rectangle highlights an irregular opaque grains outside the filaments. The red rectangle in the inset shows a close up view of a globular micrometer pyrite grain within the filament. **(D)** Photomicrograph in UV light of a circular object (dashed yellow lines). Orange rectangles highlight irregular opaque grains both inside and outside the circular object. Red rectangles highlight pyrite grains. **(E)** Detail of the clotted micrite surrounding the filament-rich patches. The white circles indicate the sites of micro-Raman analyses.

Inside the filamentous and circular objects, petrographic observations revealed the presence of opaque globular grains up to 1.5 μm across and of larger irregularly shaped opaque grains up to 10–20 μm across (Figure 5C,D). While the globular grains are present only inside the filaments and circular features (Figure 5C,D), the larger opaque grains are also found outside the filaments (Figure 5C). Both the filaments and the circular objects are embedded in a low-Mg, non-epifluorescent spar-calcite cement (Figure 5C) and are grouped together to form centimeter-sized irregular patches dispersed in a matrix of clotted micrite (Figure 4A,B). The latter is composed of aggregates of micrometer-sized peloids engulfed within a microsparitic cement (Figure 5E). Exclusively inside the irregular patches, pseudomorphs after lenticular or prismatic gypsum crystals, hundreds of micron to few millimeters in size, are common (Figure 4A, 5B). Such pseudomorphs can be empty or totally filled with sparry calcite (Figure 4A, 5B) and/or native sulfur. Finally, rod-shaped objects (Figure 4I) are present both in the patches dominated by filamentous and circular microstructures and in the clotted micrite. Under the optical and UV microscope, the distinction of these features from the filamentous and circular objects is difficult, apart for their smaller size (up to 60 μm across and 120 μm long). However, backscatter SEM imagery indicate that the rod-shaped grains contain abundant silt-sized terrigenous grains (clay and mica flakes, quartz), which are instead absent in the filamentous and circular features. On the contrary, the latter are indistinguishable in backscatter SEM observations because of the same low-Mg calcite composition as the surrounding spar cement.

### Raman Microspectroscopy

Raman spectra (Figure 5A) acquired from the microsparitic calcite inside the filamentous and circular microstructures (Figure 5C,D), from the clotted micrite (Figure 5E) and from the spar-calcite cement (Figure 5C) indicate that all these components are composed of low-Mg calcite crystals (with typical peaks at 1088, 285, 158, and 713 cm^-1^; Figure 5A, spectra b,c), in agreement with SEM-EDS data. All the spectra display a relevant fluorescence and, excluding the spectra from the spar-calcite cement (Figure 5A, spectrum b), all the other spectra show two additional broad bands (∼1350–1370 cm^-1^ and ∼1580–1600 cm^-1^; Figure 5A, spectrum c) that are indicative of poorly organized carbonaceous material, possibly finely scattered because not visible under the microscope. The ∼1580–1600 cm^-1^ band (i.e., the ordered band, also called “graphite-like” G band) is generated by the sp2 bonds typical of the crystalline carbon, whereas the ∼1350–1370 cm^-1^ band corresponds to the disordered, i.e., amorphous, D band and it is related to poor symmetry in the crystalline structure (Kelemen and Fang, 2001; Khatibi et al., 2018). The coarse-grained sparry cement filling the prismatic and lenticular pseudomorphs after gypsum (Figure 4A, 5B) shows Raman peaks at 1085, 281, 154, and 711 cm^-1^, which are typical of “pure” calcite (Figure 5A, spectrum a). With respect to the previously described spar, microspar and micritic low-Mg calcite, Raman spectra from this coarser sparry calcite do not show fluorescence and lack the two pronounced peaks of the poorly organized carbonaceous material. Spectra of pure (i.e., not mixed with calcite), poorly organized carbonaceous material (Figure 5A, spectrum e) are also obtained from the irregularly-shaped opaque grains present both inside and outside filaments and circular objects (Figure 5C,D; see section “Petrographic Observations”). On the contrary, the smaller globular opaque grains, only present within filaments and circular objects (Figure 5C,D; see section “Petrographic Observations”), have been identified as microcrystalline pyrite because of their Raman peaks at 376, 342, and 428 cm^-1^ (Figure 5A, spectrum d).

### Stable Isotopes

**FIGURE 6:**
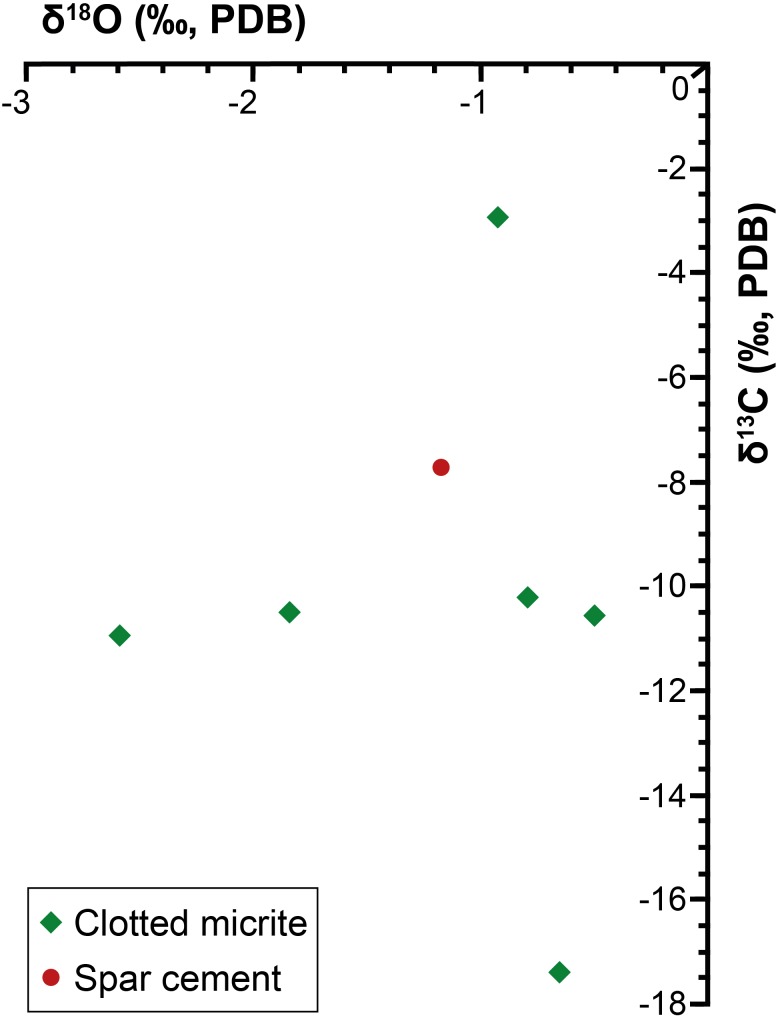
Cross-plot of the calcite stable isotope data.

Both the spar calcite forming the filament-bearing patches and the surrounding clotted micrite have been analyzed (Table 1 and Figure 4A, 6). Both these two carbonate phases are marked by negative δ^13^C values ranging from -17.4 to -10.2‰ VPDB. Only bed II owns a slightly more positive value (-2.9‰ VPDB). Also the δ^18^O values show negative values, ranging from -0.5 to -2.6‰ VPDB.

**Table 1:** Mineralogy and carbon and oxygen isotope composition of carbonate phases.

Sample	Mineralogy	Cement type	δ^18^O_V PDB_ [‰]	δ^13^C_V PDB_ [‰]
SL1 (I)	Low-Mg calcite	Clotted micrite	-0.7	-17.4
SL2 (II)	Low-Mg calcite	Spar (intra-filaments)	-1.2	-7.7
	Low-Mg calcite	Clotted micrite	-0.9	-2.9
SL3 (III)	Low-Mg calcite	Clotted micrite	-0.8	-10.2
SL4 (IV)	Low-Mg calcite	Clotted micrite	-0.5	-10.6
SL5 (V)	Low-Mg calcite	Clotted micrite	-2.6	-11
SL6 (VI)	Low-Mg calcite	Clotted micrite	-1.8	-10.5


*The roman numbers refer to the layers of sulfur-bearing limestone (SL).*

## Discussion

### Fecal Pellets vs. Microbial Filaments

Filamentous and rod-shaped morphologies similar to those found in Lorca are a common feature of carbonate (Schreiber, 1978; Guido et al., 2007; Oliveri et al., 2010; Ziegenbalg et al., 2010; Dela Pierre et al., 2012, 2014; Natalicchio et al., 2013; Caruso et al., 2015; García-Veigas et al., 2015; Perri et al., 2017) and gypsum (Vai and Ricci Lucchi, 1977; Panieri et al., 2010; Schopf et al., 2012; Dela Pierre et al., 2015) layers deposited in the Mediterranean region during the late Miocene. Their origin is, however, controversial, since they have been attributed to both fecal pellets (Schreiber, 1978; Guido et al., 2007; Natalicchio et al., 2013; García-Veigas et al., 2015), algae (Vai and Ricci Lucchi, 1977) and remains of prokaryotes such as cyanobacteria (Rouchy and Monty, 1981, 2000; Martin et al., 1984; Panieri et al., 2010) and sulfide-oxidizing bacteria (Oliveri et al., 2010; Schopf et al., 2012; Dela Pierre et al., 2012, 2014, 2015; Perri et al., 2017). To shed light on the origin of these enigmatic microstructures, and especially on the affiliation of the putative microbial fossils, two requirements must be preliminary discussed: (1) the distinction between fecal pellets and the supposed prokaryotic microfossils; (2) the validation of the criteria proving the biogenicity of microbial fossils (Schopf and Walter, 1983; Buick, 1990; Cady, 2001; Cady et al., 2003; Schopf et al., 2005; Westall, 2008; Summons et al., 2011). Some of these criteria, that have been proposed for ancient Archean, Precambrian and extraplanetary rocks (i.e., that microbial features must occur in thin sections of sedimentary or low-grade metamorphic rocks, Buick, 1990; Cady, 2001), are clearly satisfied in the studied upper Miocene sedimentary deposits and they will not further discussed. Regarding the first point, shape is not conclusive, since fecal pellets often show an elongated cylindrical shape that can be confused with filaments of bacterial origin. Considering that fecal pellets are generally larger (hundreds of microns; e.g., Wassmann et al., 1999) than most of the bacteria (generally only up to few tens of microns across; e.g., Levin and Angert, 2015), size has been previously used as a criterion to distinguish fecal pellets from bacterial remains (e.g., García-Veigas et al., 2015). However, this element is ambiguous: firstly because the size of fecal pellets is highly variable (depending on the producing organism) and secondly because some prokaryotes like colorless sulfide-oxidizing bacteria are typified by filamentous morphologies (*Beggiatoa* sp. and *Thioploca* sp.) or spherical cells (*Thiomargharita*) that can be up to 200 μm across (Schulz et al., 1999; Schulz and Jorgensen, 2001; Schulz, 2006; Teske and Nelson, 2006). The presence of carbonaceous material revealed by Raman microspectroscopy (Figure 5A, spectra c, e) and UV microscopy (Figure 4F) within the body of the filaments, which can correspond to a remnant of the original biomass of the precursor organisms (Oliveri et al., 2010; Dela Pierre et al., 2012, 2014), does not help to attribute these objects to remains of prokaryotes, as organic matter is also a component of fecal pellets. However, the nature of rod-shaped and filamentous (and circular) microstructures can be unraveled by their composition.

In fact, backscatter SEM imaging revealed a high abundance of silt-sized terrigenous grains within the rod-shaped objects than in surrounding matrix (Figure 4I), suggesting that these microstructures were not originally hollow. Considering that microbe biomass decomposes very quickly after deposition and early diagenesis, the hollowness in putative microbial microfossils is regarded as a key indicator to prove their biogenicity (Buick, 1990; Cady, 2001; Schopf et al., 2010); therefore, we suggest that rod-shaped microstructures represent fecal pellets of deposit-feeder organisms (Oliveri et al., 2010) or, alternatively, of planktonic organisms such copepods (Guido et al., 2007).

In contrast, the large filaments and the circular microstructures are filled with the same low-Mg calcite cement that is found in the surrounding matrix of the rock, making impossible their distinction with backscatter SEM imaging. Lack of compaction evidences of these microstructures indicates that the low-Mg calcite precipitated during an early diagenetic phase in hollow tubes (or spheres), originally filled with the organism biomass which was subsequently degraded by sulfate-reducing bacteria communities (see section “Mechanism of Bacteria Preservation in the Rock Record”). In addition to the hollowness requirement, the filamentous microstructures are commonly gently curved (Figure 4D), typified by a rather uniform diameter throughout their length and locally by an apparent internal segmentation (Figure 4E). The above described evidences of biogenicity (hollowness, shape, dimension and internal segmentation), together with the Raman-detected carbonaceous material (see Buick, 1990; Cady, 2001), allow to assign the studied filamentous (and circular) microstructures to prokaryotic microfossils.

### The Origin of Filamentous and Circular Microfossils

Provided that the filamentous microfossils preserved in Lorca limestones are most likely of microbial origin, their affinity is still uncertain. In particular, the question that arises is whether these objects represent remains of cyanobacteria or of sulfide-oxidizing bacteria (see for instance, Dela Pierre et al., 2012). Size and shape do not allow to distinguish between these two groups of prokaryotes, as they agree with an assignment to cyanobacteria (some oscillatoriacean cyanobacteria show individual filaments up to 100 μm across; Demoulin and Janssen, 1981), but are also consistent with colorless sulfide-oxidizing bacteria (like *Beggiatoa* and *Thioploca*), which shows filaments up to 200 μm across (Schulz and Jorgensen, 2001; Teske and Nelson, 2006). The presence of small aggregates of pyrite within most of the filaments (and circular objects) is instead noticeable. A clade diagnostic feature of living sulfide-oxidizing bacteria is the presence of zero-valent sulfur globules within the cell, which represent an intermediate product of the oxidation of hydrogen sulfide to sulfate (Nelson and Castenholz, 1981; Fossing et al., 1995; Schulz et al., 1999; Taylor et al., 1999; Schulz and Jorgensen, 2001; Wirsen et al., 2002; Schulz, 2006; Teske and Nelson, 2006). Sulfur can react with iron during early diagenesis, fostering the formation of pyrite (e.g., Peckmann et al., 2004; Bailey et al., 2009, 2013; Dela Pierre et al., 2012, 2015). In this sense, we suggest that the presence of microcrystalline pyrite grains only within the filamentous microstructures and not outside (Figure 5C,D), as revealed by Raman microspectroscopy (Figure 5A, spectrum d), possibly represents the product of the early diagenetic transformation of original sulfur globules stored by sulfide-oxidizing bacteria. Therefore, the filamentous microfossils can potentially represent fossilized sulfide-oxidizing bacteria. This interpretation is further corroborated by the curved shape of most of the filaments (Figure 4D), which is consistent with the chemotactic behavior of present-day sulfide-oxidizing bacteria (Møller et al., 1985). The origin of the circular grains is even more enigmatic. At a first glimpse these features, that are characterized by the same composition and yellow fluorescence (Figure 4G, 5D) as the filaments, as well as by the presence of the pyrite grains possibly deriving from former tiny sulfur globules, could represent cross- or transversal sections of the filamentous structures described above. In alternative, the nearly perfect rounded shape, their large diameter (up to 120 μm) and the clustering into strings up to 800 μm long (Figure 4H) may suggest remains of other “big” bacteria with similar morphological characteristics, such as those of the genus *Thiomargarita* (Schulz et al., 1999). These prokaryotes, phylogenetically close to *Beggiatoa* and *Thioploca* (Schulz, 2006), consist of spherical cells 100–750 μm across (Schulz et al., 1999; Teske and Nelson, 2006) that in modern marine settings are found isolated or grouped within a common organic membrane (Schulz et al., 1999; Schulz, 2006).

In modern settings, sulfide-oxidizing bacteria form irregular patches at the sea bottom where high fluxes of hydrogen sulfide are provided by intense bacterial sulfate reduction (BSR) in underlying organic-rich sediments (Suits and Arthur, 2000; Bailey et al., 2009; Jessen et al., 2016). The patchy arrangement of the filamentous microfossils in the studied examples is consistent with their interpretation as putative sulfide-oxidizing bacteria. A further element is their embedding in a clotted micrite (Figure 4A,B), which is considered as a product (Riding, 2000; Oliveri et al., 2010) of the degradation of organic material (bacterial cells, extracellular polymeric substances) by sulfate-reducing bacteria communities (Riding and Tomás, 2006).

### Insights on Paleoenvironmental Conditions of the Lorca Basin During the Late Miocene

**FIGURE 7:**
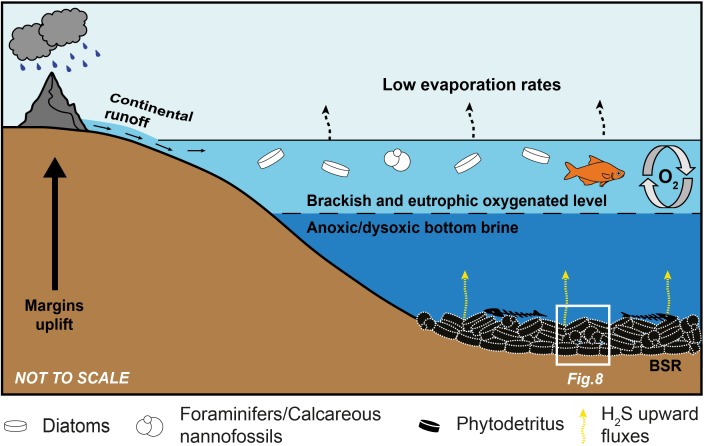
Sketch showing the paleoenvironmental conditions responsible for deposition of the precursor sediments of the sulfur-bearing limestones. See text for explanation.

**FIGURE 8:**
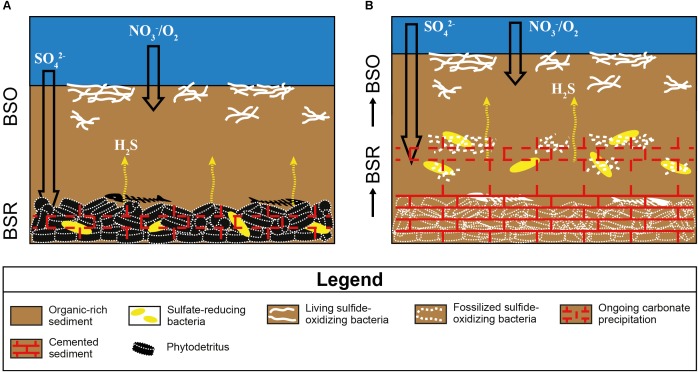
Sketch showing the origin of the sulfur-bearing limestones I, II, and V from the Lorca Basin. BSO, bacterial sulfide oxidation; BSR, bacterial sulfate reduction. **(A)** Degradation of organic matter by sulfate-reducing bacteria in bottom sediments generates an upward flux of hydrogen sulfide, which sustains the development of sulfide-oxidizing bacteria at the seafloor. The alkalinity increase promotes the early precipitation of low-Mg calcite cement. **(B)** The aggradation of the sea bottom, due to continuous deposition of organic-rich sediments, causes the upward shift of sulfate-reducing bacteria, which calcify the now buried sulfide-oxidizing bacteria.

The sulfur-bearing limestones of Lorca were considered as the product of diagenetic transformations of precursor gypsum beds formed in a shallow-water evaporitic basin (Benali et al., 1995; Russell et al., 1997; Rouchy et al., 1998). In such a scenario, carbonate precipitation was attributed to intense BSR, causing the precipitation of carbonate cement and the penecontemporaneous dissolution of the interstitially grown gypsum (Rouchy et al., 1998). However, a shallow-water evaporitic environment is in contrast with the sedimentological attributes of the Tripoli Fm., such as the evidence of synsedimentary deformations (slumps folds, Figure 3A–C) and of gravitative flows (silty-sandy chaotic layers, Figure 3D), the abundant and well preserved calcareous plankton microfossils (foraminifers and nannofossils) and open marine diatom taxa (e.g., Rouchy et al., 1998; Jurkschat et al., 2000; Krijgsman et al., 2000), which rather suggest a (relatively) open and deep marine basin. The presence of putative remains of sulfide-oxidizing bacteria does not provide useful paleobathymetrical and salinity information, since in modern environments these prokaryotes grow at different water depths (from peritidal to bathyal settings: e.g., Bailey et al., 2009; Dupraz et al., 2009) and salinity conditions (from freshwater to hypersaline conditions; Teske and Nelson, 2006; Perri et al., 2017). However, the identification of these prokaryotes in the fossil record could be particularly important to trace the presence of bottom sulfidic conditions (e.g., Jessen et al., 2016). Living sulfide-oxidizing bacteria are reported from sites where high fluxes of hydrogen sulfide, which is oxidized to elemental sulfur and sulfate (Kuenen, 1975; Wood and Kelly, 1986; Van Gemerden, 1993; Schulz and Jorgensen, 2001; Kamp et al., 2006; Teske and Nelson, 2006; Bailey et al., 2009; Jessen et al., 2016), are provided by intense BSR in underlying organic-rich sediments, such as hydrothermal vents (Sievert et al., 2008) and cold seeps (Himmler et al., 2018), and in other oxygen-depleted waters (e.g., the hypoxic zone of the Black Sea, Pilskaln and Pike, 2001; Jessen et al., 2016; the continental shelf off the Peru coast, Suits and Arthur, 2000; the Santa Barbara basin, Reimers et al., 1990). Under oxygen-depleted conditions, some bacteria can use nitrate as an electron acceptor for hydrogen sulfide oxidation (Van Gemerden, 1993; Teske and Nelson, 2006; Bailey et al., 2009; Dupraz et al., 2009). In Lorca, the sedimentological features of the Lower Member of the Tripoli Fm., which hosts the studied authigenic limestones, agree with the occurrence, at least temporarily, of a stratified water column typified by high primary productivity in the upper layers, which can account for the widespread deposition of biosiliceous deposits in a basin affected by ongoing restriction (Rouchy et al., 1998; Jurkschat et al., 2000; Krijgsman et al., 2000). Most likely, stratification and productivity were triggered by the input of continental freshwater during humid climate phases (Figure 7), which agrees with the negative δ^18^O values of all the samples. These conditions have favored the spread of oxygen-depleted bottom conditions and organic matter accumulation (Rouchy et al., 1998; Figure 7), as further suggested by the lack of bioturbation in the diatomaceous sediments and the very high total organic carbon content of the shales associated to the sulfur-bearing limestones (up to 22%; see Permanyer et al., 1991, 1994) in various boreholes (IGME, 1982) and in old sulfur mines (Permanyer et al., 1991, 1994). Eutrophication and hypoxia most likely favored intense BSR in bottom sediments and sustained the growth of sulfide-oxidizing bacteria communities at the sea floor (Figure 8A).

### Mechanism of Bacteria Preservation in the Rock Record

The preservation of sulfide-oxidizing bacteria in the geological record can take place thanks to the rapid (and early) precipitation of authigenic carbonate necessary for the entombment of the delicate organic tissues of these prokaryotes before their complete decay (e.g., Bailey et al., 2009). However, the metabolism of sulfide-oxidizing bacteria is in apparent contrast with carbonate precipitation, since sulfide oxidation promotes an increase of acidity more favorable to the dissolution of carbonate, if the reaction occurs with oxygen as electron acceptor (e.g., Peckmann et al., 2004; Bailey et al., 2009; Petrash et al., 2012; Himmler et al., 2018). Nitrate-driven sulfide oxidation, which is performed by representative of the genus *Thioploca*, can instead favor an alkalinity rise and carbonate precipitation as observed in calcified microbial mats from the Northern Arabian sea (Himmler et al., 2018). Our data do not permit to discriminate among nitrate or oxygen as electron acceptor used for the reaction of sulfide oxidation. However, the negative δ^13^C values (-17.4 < δ^13^C < -10.2‰ VPDB) of both the spar cement that entombs the filaments and circular microfossils and the clotted micrite (Table 1 and Figure 4A, 6), are typical of microbial carbonates (Riding, 2000; Riding and Tomás, 2006; Oliveri et al., 2010) and are fully consistent with authigenic carbonate precipitation triggered by BSR (Machel, 2001; Baumgartner et al., 2006; Jørgensen and Kasten, 2006; Ziegenbalg et al., 2010); in turn, BSR provided the hydrogen sulfide flux exploited by sulfide-oxidizing prokaryotes at the sea bottom (Figure 8A). We suggest that the continuous accumulation of organic-rich sediments at the sea floor caused the upward shift of bacterial communities, giving rise to precipitation of carbonate cement via BSR and favoring the entombment of the now buried prokaryotes (Figure 8B). According to this scenario, sulfide oxidation (possibly promoting acidity) and sulfate reduction (promoting carbonate precipitation) were spatially and temporarily decoupled, allowing the preservation of sulfide-oxidizing bacteria in the rock record (Figure 8B).

## Conclusion

Three out of six levels of sulfur-bearing limestones interlayered to the upper Miocene diatomaceous sediments from the Lorca Basin are mostly composed of fecal pellets (rod-shaped microstructures) and microbial microfossils (filamentous and circular microstructures) representing putative sulfide-oxidizing bacteria. The development of chemotrophic microbial communities at the sea bottom is the result of complex biogeochemical cycles involving both sulfate reduction and sulfide oxidation. Organic matter degradation by sulfate-reducing bacteria in the subsurface ensured an incessant flow of H_2_S required for the proliferation at the sea bottom of sulfide-oxidizing bacteria communities and induced the precipitation of the low-Mg calcite spar cement, allowing their preservation in the fossil record. These metabolic processes were favored by the oxygen-depleted sea bottom conditions of the basin, in turn related to salinity stratification of the water column induced by the supply of continental freshwater. Even though these bacteria are light independent, they do not provide any paleobathymetric information, because they are adapted to live at any depth. However, sedimentological and paleontological features of the sediments hosting the carbonates levels in which they are found point to a relatively deep marine environment.

## Author Contributions

FA conducted the field and laboratory work, and wrote the manuscript. FD and LG supervised the research and contributed to the writing of the manuscript. MN contributed to the discussion. SF provided the micro-Raman analyses and their interpretation. All authors revised the work critically and approved the manuscript.

## Conflict of Interest Statement

The authors declare that the research was conducted in the absence of any commercial or financial relationships that could be construed as a potential conflict of interest.
